# Ultrasound Versus Age-Based Formula for Predicting Endotracheal Tube Size in Pediatric Patients Undergoing General Anesthesia: A Randomized Comparative Study

**DOI:** 10.7759/cureus.102359

**Published:** 2026-01-26

**Authors:** Syama Sundar Ayya, Anish Waghray, Aniruth Chand Anna C, Rama Krishna Prasad Chikkala, Sandeep Garre

**Affiliations:** 1 Anesthesiology, All India Institute of Medical Sciences, Bibinagar, Bibinagar, IND; 2 Pain Medicine, Amista Hospital and Clinics, Hyderabad, IND

**Keywords:** endotracheal intubation, endotracheal tube size prediction, pediatric intubation, placement of endotracheal tube (ett), ultrasound-guided

## Abstract

Introduction

Selection of an appropriately sized endotracheal tube (ETT) in pediatric patients is critical to ensure adequate ventilation and minimize airway-related complications. Age-based formulae are commonly used but often fail to account for inter-individual anatomical variability. Ultrasonographic assessment of the subglottic airway offers a patient-specific alternative that may aid in selecting the optimal ETT size. This study compared ultrasound-guided estimation of ETT size with the age-based formula (internal diameter = age/4 + 3.5) and evaluated their agreement with the clinically optimal ETT size.

Methods

This prospective, randomized, comparative study was conducted at a tertiary care center and included children aged six months to 12 years undergoing elective surgery under general anesthesia. Participants were randomized to either an ultrasound group or a formula group. In the ultrasound group (Group U), subglottic airway diameter was measured at the cricoid level by ultrasonography, and ETT size was determined by subtracting 0.5 mm from the subglottic diameter. In the formula group (Group F), ETT size was calculated using an age-based formula. The clinically optimal ETT size determined using the leak pressure technique served as the reference standard. Agreement between predicted and used ETT sizes and the requirement for tube exchange were analyzed.

Results

After exclusions, 137 patients were included in the final statistical analysis (Group F, n = 70; Group U, n = 67). Baseline demographic characteristics were comparable between groups. Ultrasound-guided estimation resulted in a significantly lower incidence of oversized ETT selection (Group U vs F; 8 (11.9%) vs. 20 (28.6%), P = 0.045). The incidence of ETT exchange decreased in the ultrasound group, although this did not reach statistical significance. The Bland-Altman analysis confirmed superior agreement in the ultrasound group, with a smaller mean bias (-0.0448 mm vs. -0.129 mm) and lower percentage error (11.5% vs. 13.4%) compared with the formula group. No airway-related complications were observed in either group.

Conclusion

Ultrasound-guided measurement of the transverse subglottic diameter is superior to the age-based formula for ETT size selection in children. It provides a patient-specific anatomical assessment that significantly improves prediction accuracy and reduces the risk of oversized ETT selection.

## Introduction

Appropriate selection of endotracheal tube (ETT) size is a fundamental skill in pediatric anesthesia. It is essential to secure the airway safely without causing trauma and to reduce the need for repeated intubation attempts. Undersized ETTs may lead to inadequate ventilation, aspiration, and leakage of anesthetic gases, while oversized ETTs increase the risk of laryngeal oedema and tracheal mucosal injury [1,2]. Traditionally, age-based formulae have been used to estimate ETT size, with Cole's formula (internal diameter (ID) = age/4 + 4) being widely accepted for uncuffed ETTs. Over time, technological advances have made cuffed ETTs safer and more effective, even in the pediatric population. They ensure a good seal and reduce the need for multiple tube changes, which lowers the risk of airway-related injuries [3,4]. For cuffed ETTs, Duracher's formula (ID = age/4 + 3.5) has been shown to provide a more appropriate estimate [5]. Its accuracy is often compromised by inter-individual anatomical variability and manufacturing variations, where tubes with the same ID may have various outside diameters, often resulting in suboptimal tube selection [6]. To address these limitations, alternative sizing methods, including magnetic resonance imaging (MRI), ultrasonography, and three-dimensional airway modelling, have been explored to improve the accuracy of pediatric ETT size estimation [7,8]. Among these, ultrasonography offers a noninvasive, bedside technique that allows direct measurement of the subglottic airway diameter [9]. However, only a few studies have directly compared ultrasound-guided selection with Duracher's age-based formulae for initial ETT placement [10]. Therefore, the present study was designed to compare ultrasound-guided estimation of ETT size with age-based (Duracher's) formula and to evaluate their agreement with the clinically optimal tube size determined using the leak pressure technique.

## Materials and methods

This was a prospective, randomized comparative study conducted at a tertiary care teaching hospital. The study was approved by the Institutional Ethics Committee, AIIMS Bibinagar (AIIMS/BBN/IEC/JUNE/2023/281-R). The trial was prospectively registered with the Clinical Trials Registry of India (CTRI/2024/02/062295). Written informed consent was obtained from parents or legal guardians prior to enrolment. The study included children aged six months to 12 years, with an American Society of Anesthesiologists (ASA) physical status of I or II, scheduled for elective surgery under general anesthesia [11]. Patients were excluded if they presented with craniofacial or airway anomalies, laryngeal or tracheal pathology, recent upper respiratory tract infections, or required emergency surgery.

On the day of surgery, all participants were maintained nil per os, according to standard fasting guidelines, with a minimum fasting period of six hours for light meals, four hours for mothers' milk, and two hours for clear liquids prior to induction of anesthesia. All eligible patients were randomly allocated to two groups of 70 each: Group U (ultrasound) and Group F (formula). Randomization was performed using a computer-generated randomization sequence. Allocation concealment was ensured using sequentially numbered, opaque, sealed envelopes (SNOSE) prepared by an independent investigator not involved in patient enrolment or data collection. Envelopes were opened only after enrolment and immediately before induction of anesthesia. Blinding of the anesthesiologist was not feasible due to the nature of the intervention.

**Figure 1:**
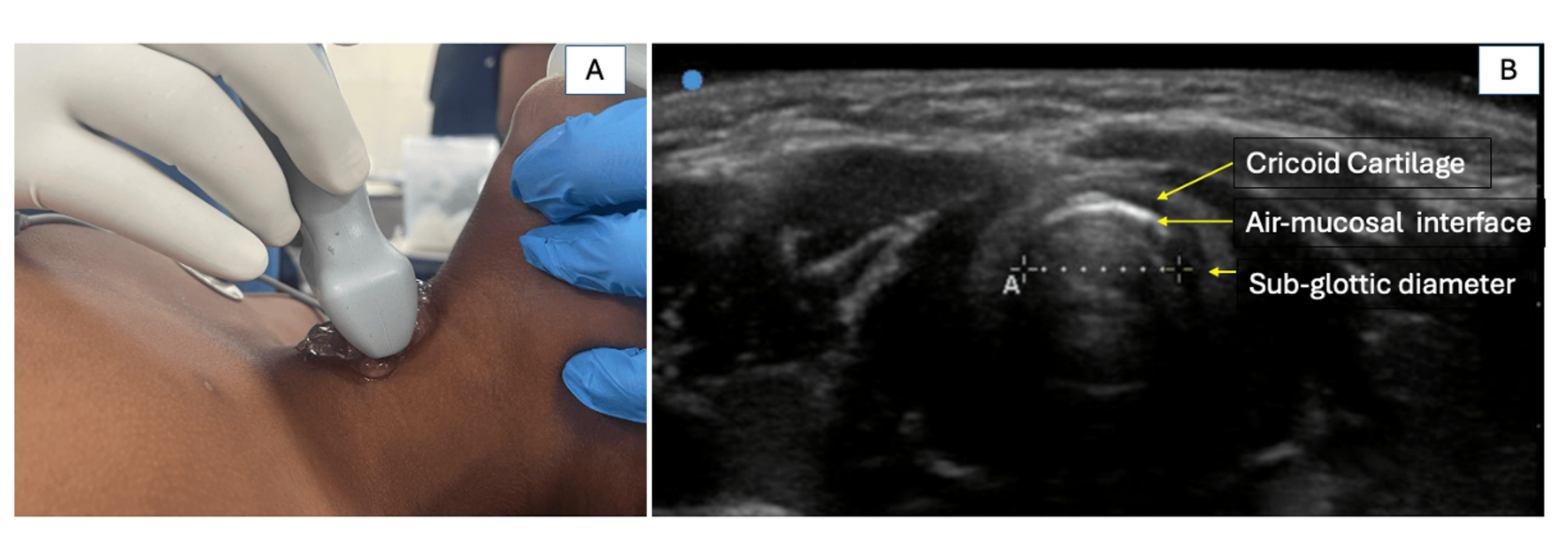
Ultrasonographic measurement of the transverse subglottic diameter at the level of the cricoid cartilage. (A) Patient position and placement of the high-frequency linear ultrasound probe over the anterior central region of the neck. (B) Transverse subglottic diameter was measured as the distance between the air-mucosa interfaces (marked by calipers) within the inner margins of the cricoid cartilage.

After shifting to the operating room, noninvasive monitoring, including electrocardiography (ECG), noninvasive blood pressure (NIBP), and pulse oximetry, was applied to all patients. Anesthesia was induced with either an inhalational or an intravenous agent (sevoflurane or propofol, 1-2 mg/kg), depending on the availability of intravenous access and at the attending anesthesiologist's discretion, and neuromuscular blockade was achieved with atracurium 0.5 mg/kg. After achieving adequate neuromuscular relaxation, the trachea was intubated with an ETT (Sterimed Medical Devices Pvt. Ltd., New Delhi, India) under direct laryngoscopy. In the formula group, ETT size was selected using a Duracher's age-based formula (ID = age/4 + 3.5) for cuffed tubes in children aged six months to 12 years. In the ultrasound group, subglottic airway transverse diameter was measured before laryngoscopy by an anesthesiologist with more than three years of experience in ultrasound-guided airway assessment, using a high-frequency linear probe (6-13 MHz) in B-mode (Sonosite Edge II, Fujifilm Sonosite Inc., Bothell, WA, USA). The probe was placed on the midline of the anterior neck, with the child in the supine position and slight neck extension while ventilated via facemask. The examination began with identification of the true vocal cords to avoid confusion with tracheal rings, and the subglottic diameter was measured at the level of the cricoid cartilage as the transverse diameter of the hyperechoic air column (Figure 1).

Adequate ultrasound images were successfully obtained in all patients, allowing measurement of the subglottic diameter in every case. To avoid an overly snug fit, 0.5 mm was subtracted from the measured diameter to closely approximate the final ETT outer diameter (OD).

The clinically optimal ETT size was determined using the leak pressure technique, with leak pressure assessed by placing a stethoscope over the suprasternal notch while observing airway pressure before cuff inflation [12,13]. A leak detected at an inflation pressure between 10 and 25 cm H_2_O was considered optimal. If a leak occurred at a pressure < 10 cm H_2_O, the tube was exchanged for one 0.5 mm larger; if the leak was detected at a pressure > 25 cm H_2_O, the tube was exchanged for one 0.5 mm smaller at the discretion of the treating anesthesiologist. After surgery, patients were extubated and monitored in the post-anesthesia care unit. All patients were monitored for 24 hours postoperatively for respiratory complications such as stridor, laryngospasm, hypoxia, or the need for reintubation.

The primary objective of this study was to compare the accuracy of ultrasonography and age-based (Duracher's formula) in selecting an appropriately sized ETT on the first attempt. The secondary objectives were to compare the incidence of ETT exchange and to evaluate the frequency of oversized and undersized tube predictions in both groups.

The sample size for this study was determined based on a comparison of proportions between two independent groups. Based on the study by Gnanaprakasam and Selvaraj, in which the incidence of appropriate ETT selection was 45% with age-based formulae, an absolute difference in proportions of 25% between the formula and ultrasound groups was considered clinically significant [10,14]. With a two-sided alpha error of 0.05 and a statistical power of 80%, the minimum required sample size was calculated as 66 participants per group. To compensate for potential dropouts, the recruitment target was increased to 140 patients, with 70 patients allocated to each group.

Statistical analysis

Statistical analysis was performed using Jamovi statistical software (version 2.6; The Jamovi Project, Sydney, Australia). Continuous variables were expressed as mean ± standard deviation and median (interquartile range), and categorical variables were presented as frequencies and percentages. The Shapiro-Wilk test was used to assess the normality of the data. Continuous variables, such as age, weight, and the number of tubes used per patient, were compared between groups using the Mann-Whitney U test. Categorical variables were analyzed using the chi-square test or Fisher's test, as appropriate. Within-group comparisons between predicted and clinically used ETT IDs were performed using the Wilcoxon signed-rank test. Agreement between the predicted and used ETT sizes within each group was assessed using a Bland-Altman analysis. P < 0.05 was considered statistically significant.

## Results

**Figure 2:**
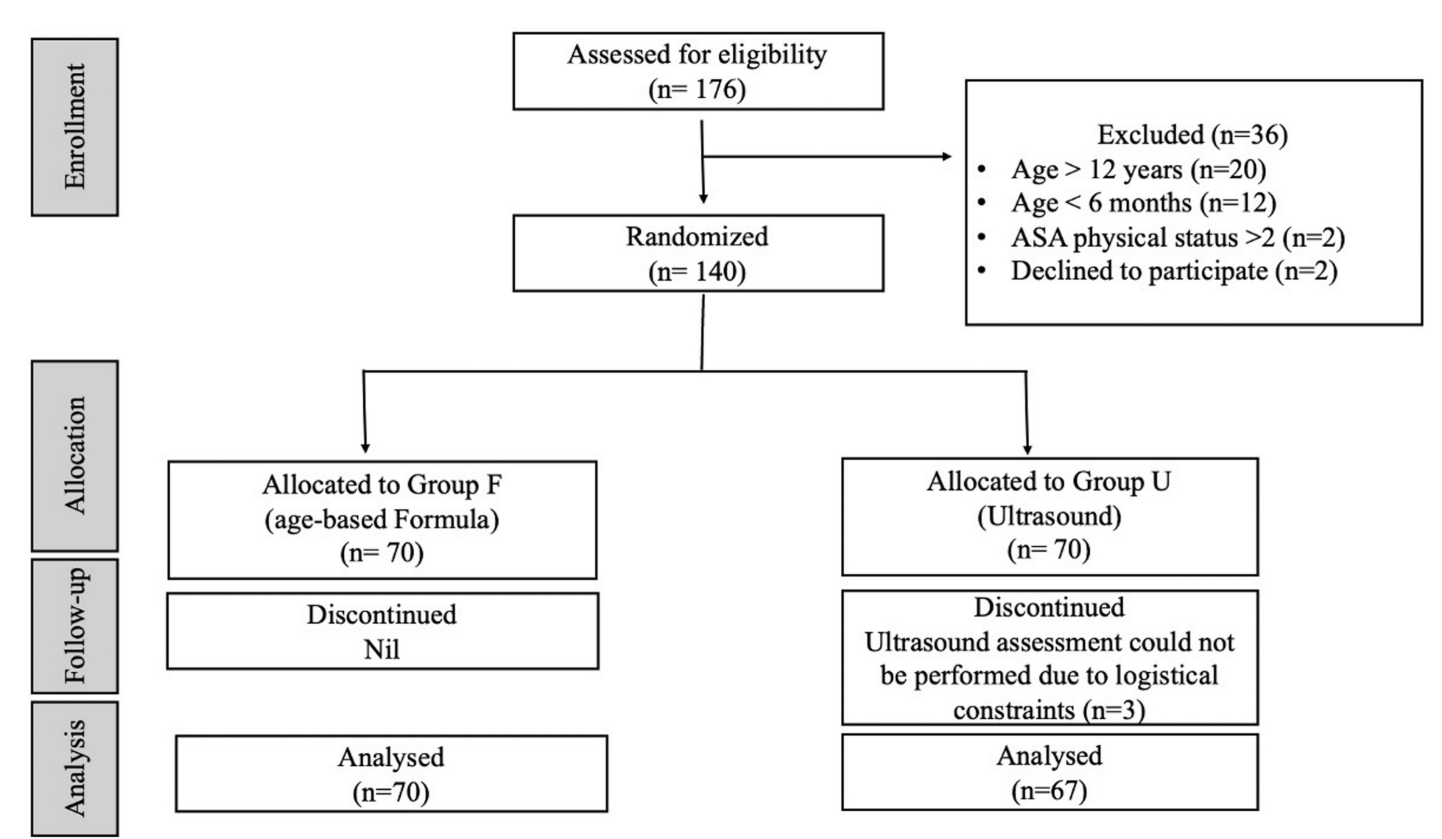
Consolidated standard of reporting trials (CONSORT) flow chart. ASA Physical Status: American Society of Anesthesiologists Physical Status Classification System [11].

Of the 140 children enrolled, 137 completed the study and were included in the final analysis, with 70 patients in the formula group (Group F) and 67 in the ultrasound group (Group U) (Figure 2).

Baseline demographic characteristics, including age, sex distribution, and body weight, were comparable between the two groups, with no statistically significant differences observed (Table 1).

**Table 1:** Characteristics of the study population. Data are presented as median (interquartile range (IQR)) or as number (percentage). Age and weight between groups were compared using the Mann-Whitney U test. Chi-square test was used to compare the gender distribution between groups. Group F: formula group, Group U: ultrasound group

Variable	Group F (n = 70)	Group U (n = 67)	Test statistic	P-value
	Median (IQR)/n (%)	Median (IQR)/n (%)		
Age (years)	5 (2-8)	4.5 (2-7.5)	U = 2257	0.636
Female	17 (25)	15 (22.1)	χ^2^ = 0.068	0.686
Male	51 (75)	53 (77.9)		
Weight (kg)	14.4 (10.2-19.3)	15.5 (10.5-20)	U = 2307	0.860

The mean number of ETTs used per patient was similar in both groups, with a trend toward fewer ETTs in the ultrasound group. Ultrasound-guided assessment resulted in a significantly lower incidence of oversized ETT selection compared with the age-based formula. The proportion of exact tube size predictions was higher in the ultrasound group. Undersized tube selection was infrequent in both groups and did not differ significantly (Table 2).

**Table 2:** Comparison of predicted and used ETT sizes between groups. Data are presented as median (interquartile range (IQR)) or as number (percentage). The number of ETT used was compared using the Mann-Whitney U test. The number of patients required ETT change, incidence of overestimation (size used < size predicted), and exact prediction of ETT size (size used = size predicted) were compared using the chi-square test. Underestimation of ETT size (size used < size predicted) was compared with Fisher’s exact test. ETT: endotracheal tube, Group F: formula group, Group U: ultrasound group

Variable	Group F (n = 70)	Group U (n = 67)	Test statistic	P-value
	Median (IQR)/n (%)	Median (IQR)/n (%)		
Number of ETT used per patient	1 (1-2)	1 (1-1)	U = 2041	0.08
Overestimation of ETT size (size used < size predicted)	20 (28.6)	8 (11.9)	χ^2^ = 5.44	0.02
Underestimation of ETT size (size used < size predicted)	3 (4.3)	5 (7.5)	-	0.724
Exact prediction of ETT size (size used = size predicted)	47 (67.1)	54 (80.6)	χ^2^ = 3.20	0.074
Number of patients required for ETT change	23 (32.9)	13 (19.4)	χ^2^ = 3.20	0.074

When comparing predicted and clinically used ETT sizes within each group, a statistically significant difference was observed in Group F, with the age-based formula tending to overestimate the required tube size (Table 3).

**Table 3:** Comparison between predicted and used ETT’s size (internal diameter (ID)) in Group F and Group U. Data are presented as median (interquartile range (IQR)). Within-group comparisons between predicted and clinically used ETT IDs were performed using the Wilcoxon signed-rank test. ETT: endotracheal tubes, Group F: formula group, Group U: ultrasound group

Variable	Predicted ETT size	Used ETT size	Test statistic	P-value
	Median (IQR)	Median (IQR)		
Group F (n = 70)	4.5 (4-5.38)	4.5 (4-5)	W = 233	0.002
Group U (n = 67)	4.5 (4-5)	4 (3.5-5)	W = 22	0.166

In contrast, no significant difference was found between predicted and used tube sizes in the ultrasound group, indicating closer agreement between ultrasound-based prediction and the clinically optimal tube size.

Agreement between predicted and clinically optimal ETT sizes was assessed using Bland-Altman analysis (Table 4).

**Table 4:** Bland-Altman plots showing agreement between predicted and used endotracheal tube sizes in Group F (formula group) and Group U (ultrasound group).

Variable	Group F (n = 70) (predicted vs. used endotracheal tube size)	Group U (n = 67) (predicted vs. used endotracheal tube size)
Mean bias (mm)	-0.129	-0.044
Standard deviation	0.31	0.25
Limits of agreement (95% confidence intervals)	-0.746 to 0.489	-0.548 to 0.459
Percentage error	13.4%	11.5%

**Figure 3:**
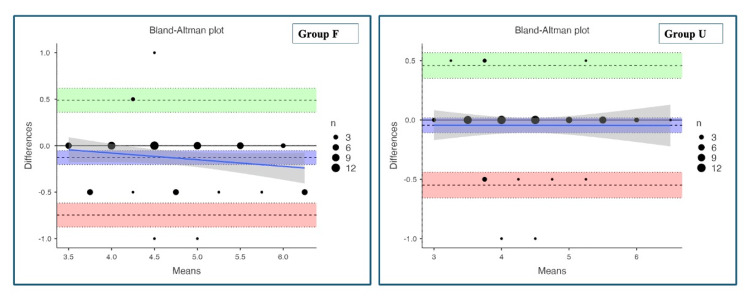
Bland-Altman plots showing agreement between predicted and used endotracheal tube sizes. The plots depict the differences between predicted and clinically used endotracheal tube sizes plotted against their means. The central solid horizontal line represents the mean bias, while the dashed horizontal lines indicate the 95% limits of agreement. The solid sloping line represents the proportional bias (regression) line with its 95% confidence band. Overlapping data points reflect multiple observations with identical mean and difference values; point size corresponds to the number of overlapping observations.

In the formula group, the mean bias was -0.129 mm with wider limits of agreement, indicating a tendency toward overestimation and greater variability (Figure 3).

The percentage error for the formula-based prediction was 13.4%. In contrast, the ultrasound group demonstrated a smaller mean bias (-0.045 mm), narrower limits of agreement, and a lower percentage error of 11.5%, reflecting improved agreement and consistency. Examination of the proportional bias line in the formula group shows a slight downward slope, suggesting a trend toward increasing disagreement with larger tube sizes. Although this trend is modest, it indicates the presence of mild proportional bias, with greater overestimation occurring at higher mean ETT sizes (Figure 3). The proportional bias line in the ultrasound group is nearly horizontal, with no apparent relationship between the magnitude of differences and the mean ETT size (Figure 3). This finding indicates the absence of proportional bias, suggesting that ultrasound-guided prediction maintains consistent agreement across the full range of tube sizes.

These findings indicate that although both methods demonstrated reasonable agreement with the clinically optimal tube size, ultrasound-guided estimation showed superior agreement and consistency compared with Duracher's age-based formula. None of the patients developed stridor, laryngospasm, hypoxia, or required reintubation during the study period.

## Discussion

In this prospective randomized study, we compared ultrasound-guided estimation of ETT size with Duracher's formula. We evaluated their agreement with the clinically optimal tube size determined by the leak pressure technique. The principal findings of our study are that ultrasound-guided assessment resulted in fewer oversized tube selections and superior agreement with the clinically optimal ETT size, as demonstrated by Bland-Altman analysis.

The improved performance of ultrasound-guided estimation may be due to direct visualization and measurement of the subglottic airway, the narrowest portion of the pediatric airway [4,9]. On the other hand, age-based formulae depend on the generalized relationships between chronological age and airway dimensions, which fail to account for individual anatomical variability. This issue is highlighted by the work of Al-Mazrou et al., who used MRI to demonstrate that the correlation between age and optimal tube size is only moderate [7]. Their research revealed that the OD of the clinically determined "best-fit" ETT was consistently smaller than the actual internal transverse diameter of the cricoid by 0.1-1.7 mm. In addition, variations in ETT design and manufacturing, especially differences in OD among brands, further limit the reliability of age-based predictions, as reported by Wankijcharoen et al. [6].

Another key clinically relevant finding was the significantly lower incidence of oversized ETT selection in the ultrasound group compared with the formula group. Oversized tubes are of particular concern in pediatric anesthesia, as they can exert excessive pressure on the delicate laryngeal and tracheal mucosa, increasing the risk of post-extubation stridor, laryngeal oedema, and long-term airway injury [15]. Our findings are consistent with those of Gnanaprakasam and Selvaraj and Altun et al., who also reported improved first-attempt tube selection and reduced oversizing with ultrasound-guided airway assessment [10,14].

When predicted and used ETT sizes were compared within each group, Duracher's age-based formula showed a statistically significant difference, indicating systematic overestimation of the required tube size. In contrast, ultrasound-based predictions did not differ significantly from the clinically optimal tube size, supporting the ability of ultrasonography to provide patient-specific estimation based on actual airway anatomy rather than population-derived averages.

Although the reduction in the total number of ETT exchanges did not reach statistical significance in our cohort (19.4% in Group U vs. 32.9% in Group F; P = 0.074), the mean number of ETT used per patient was lower in Group U than in Group F (1.21 ± 0.44 vs. 1.33 ± 0.47; P = 0.08). This reduction in ETT exchanges may be clinically important, as minimizing trial-and-error during airway instrumentation is crucial for ensuring paediatric patient safety during the vulnerable induction phase.

The Bland-Altman analysis demonstrated superior agreement with ultrasound-guided prediction compared with Duracher's age-based formula. In the formula group, consistent negative mean bias was observed, with wide limits of agreement. This indicated greater variability in prediction accuracy. Examination of the proportional bias line showed a mild negative slope, indicating greater overestimation with larger tube sizes (Figure 3). Although modest, this proportional bias is clinically relevant, as it demonstrates that formula-based predictions may become less reliable in older or larger children.

In contrast, the ultrasound group demonstrated minimal mean bias, narrower limits of agreement, and a lower percentage error (11.5% compared with 13.4% in the formula group). The proportional bias line in the ultrasound group was nearly horizontal, indicating the absence of size-dependent bias and consistent agreement across the full range of ETT sizes studied. The lower percentage error observed with ultrasound emphasizes its superior precision and reliability.

Despite these advantages, our study has certain limitations. Our study methodology relied on measurement of the transverse diameter of the subglottic airway. As noted in the literature, the pediatric airway is often elliptical, with an anteroposterior (vertical) diameter that is frequently larger than the transverse diameter [16,17]. Blinding of the anesthesiologist was not feasible due to the nature of the intervention. However, objective leak pressure criteria were used to minimize bias in determining the clinically optimal ETT size. Ultrasound assessment is operator-dependent and requires training and experience, which may limit generalizability. This study was conducted at a single center, and multicenter trials may be needed to confirm these findings across different practice settings.

## Conclusions

Sonographic measurement of the subglottic diameter at the cricoid level provides an accurate and precise method for ETT size selection in children. It significantly reduces the risk of selecting oversized ETTs compared to Duracher's age-based formula. These findings support the role of ultrasonography as a valuable adjunct for pediatric ETT size selection.

## References

[1] Gomes Cordeiro AM, Fernandes JC, Troster EJ (2004). Possible risk factors associated with moderate or severe airway injuries in children who underwent endotracheal intubation. Pediatr Crit Care Med.

[2] Fonseca JG, de Moura CF, Rézio GS (2025). Risk factors and clinical outcomes of post-extubation stridor in pediatric intensive care. Children (Basel).

[3] Chambers NA, Ramgolam A, Sommerfield D (2026). Cuffed vs. uncuffed tracheal tubes in children: a randomised controlled trial comparing leak, tidal volume and complications. Anaesthesia.

[4] Tobias JD (2015). Pediatric airway anatomy may not be what we thought: implications for clinical practice and the use of cuffed endotracheal tubes. Paediatr Anaesth.

[5] Duracher C, Schmautz E, Martinon C, Faivre J, Carli P, Orliaguet G (2008). Evaluation of cuffed tracheal tube size predicted using the Khine formula in children. Paediatr Anaesth.

[6] Wankijcharoen J, Khamman P, Thusneyapan K (2025). Characteristics of endotracheal tube design of different brands are related to proper endotracheal tube position in pediatrics: a descriptive study. Transl Pediatr.

[7] Al-Mazrou KA, Abdullah KM, Ansari RA, Abdelmeguid ME, Turkistani A (2009). Comparison of the outer diameter of the 'best-fit' endotracheal tube with MRI-measured airway diameter at the cricoid level. Eur J Anaesthesiol.

[8] Park S, Ahn J, Yoon SU, Choo KS, Kim HJ, Chung M, Kim HY (2021). Prediction of endotracheal tube size using a printed three-dimensional airway model in pediatric patients with congenital heart disease: a prospective, single-center, single-group study. Korean J Anesthesiol.

[9] Seo H, Oh CS, Choi GJ, Park JB (2025). Clinical roles of point-of-care ultrasonography in airway management. Anesth Pain Med (Seoul).

[10] Altun D, Sungur MO, Ali A, Bingül ES, Seyhan TÖ, Çamcı E (2016). Ultrasonographic measurement of subglottic diameter for paediatric cuffed endotracheal tube size selection: feasibility report. Turk J Anaesthesiol Reanim.

[11] (2026). American Society of Anesthesiologists statement on ASA physical status classification system. Anesthesiology Open.

[12] Bharathi BM, Somayaji S, Tulasi T, Sheriff NK, Bagliker JS (2022). Prediction of endotracheal tube size in pediatric population using ultrasonographic subglottic diameter and age-related formulas: a comparative study. Anesth Essays Res.

[13] Sutagatti JG, Raja R, Kurdi MS (2017). Ultrasonographic estimation of endotracheal tube size in paediatric patients and its comparison with physical indices based formulae: a prospective study. J Clin Diagn Res.

[14] Gnanaprakasam PV, Selvaraj V (2017). Ultrasound assessment of subglottic region for estimation of appropriate endotracheal tube size in pediatric anesthesia. J Anaesthesiol Clin Pharmacol.

[15] Park S, Shin SW, Kim HJ, Byeon GJ, Yoon JU, Kim EJ, Kim HY (2022). Choice of the correct size of endotracheal tube in pediatric patients. Anesth Pain Med (Seoul).

[16] Griscom NT, Wohl ME (1986). Dimensions of the growing trachea related to age and gender. AJR Am J Roentgenol.

[17] Litman RS, Weissend EE, Shibata D, Westesson PL (2003). Developmental changes of laryngeal dimensions in unparalyzed, sedated children. Anesthesiology.

